# Duration and clinical outcomes of dual antiplatelet therapy following percutaneous coronary intervention for acute coronary syndrome: A multicentre “real-world practice” registry-based study

**DOI:** 10.3389/fcvm.2023.1158466

**Published:** 2023-04-06

**Authors:** Carlos E. Vergara-Uzcategui, Víctor H. Moreno, Breda Hennessey, Rafael Sánchez-del-Hoyo, José H. Donis, Jorgelys Gonzalez-Rojas, Pablo Salinas, Luis Nombela-Franco, Nieves Gonzalo, Pilar Jimenez-Quevedo, Hernán Mejia-Renteria, Javier Escaned, Antonio Fernández Ortiz, Carlos Macaya Miguel, Iván J. Núñez-Gil

**Affiliations:** ^1^ Cardiovascular Institute, Hospital Clínico San Carlos, IdISSC, Madrid, Spain; ^2^ Faculty of Medicine, Universidad Complutense de Madrid, Madrid, Spain; ^3^ Research methodological support unit and Preventive Department, Hospital Clínico San Carlos, IdISSC, Madrid, Spain; ^4^ Faculty of Medicine, Universidad de Los Andes, Mérida, Venezuela; ^5^ Faculty of Biomedical and Health Sciences, Universidad Europea de Madrid, Villaviciosa de Odón, Spain

**Keywords:** dual antiplatelet therapy, duration, outcomes, acute coronary syndrome, percutaneous coronary intervention

## Abstract

**Background:**

The optimal duration of dual antiplatelet therapy (DAPT) ought to be determined taking into account individual ischaemic or bleeding events risks. To date, studies have provided inconclusive evidence on the effects of prolonged DAPT. We sought to evaluate the long-term outcomes of this strategy following percutaneous revascularization in the context of acute coronary syndrome (ACS).

**Methods:**

Retrospectively from four centers in Madrid, we identified 750 consecutive ACS patients, divided in two groups of DAPT duration: <13 months and >13 months, with a mean follow-up of 48 months.

**Results:**

Patients with DAPT > 13 months had a higher non-adjusted incidence of Major Adverse Cardiovascular Events (11.6% vs. 17.3%) and new revascularization (3.7% vs. 8.7%). Differences in all-cause death, cardiac death, myocardial infarction, stent thrombosis and stroke were non-significant. There was no difference in the incidence of major bleeding (7.4% vs. 6.3%). Multivariable Cox regression analysis showed that the independent risk predictors of MACE were age (HR: 1.04, 95% CI: 1.02–1.06, *p* < 0.001) and multivessel disease (HR: 2.29, 95% CI: 1.32–3.95, *p* = 0.003), whereas the independent protective predictor was normal hemoglobin (HR: 0.88, 95% CI: 0.78–0.98, *p* = 0.022).

**Conclusions:**

In this real-world registry cohort of ACS patients treated with PCI and 1 year of DAPT in Spain, we report a trend of increased rate of MACE and new revascularization not associated with TVR in patients with longer DAPT. Our findings support the need for future randomized controlled trials to confirm or refute these results.

## Introduction

The optimal duration of dual antiplatelet therapy (DAPT) in patients undergoing percutaneous coronary intervention (PCI) for acute coronary syndrome (ACS) is still being investigated. DAPT duration ought to be determined based on the patient characteristics, the association with ischaemic or bleeding events risks and the procedural characteristics which includes type of lesion, stent type and length and the use of intravascular imaging to optimise the procedural result (1–4). Studies reporting optimal DAPT duration provide inconclusive evidence on the effect of the prolonged DAPT strategy on all-cause mortality, although some reports show a trend towards an increased risk (5). Furthermore, real-world experience data on the type, duration, rate, and predictors of long-term DAPT indication are lacking and, in some cases, controversial (6).

Patients with ACS, are thought to be at a higher risk of ischaemic events, and benefit from a longer duration of DAPT (1). The most significant risk period for ischaemic events is during the first year following an ACS, during which the current guidelines recommend 12 months of DAPT for patients with a lower bleeding risk. It is known that cardiac or cerebrovascular ischaemic events, such as cardiac death, myocardial infarction (MI), or stroke, that are unrelated to the treated culprit lesion can occur as well beyond 12 months, and there remains a residual risk of these events (about 20%) during the first four years of follow-up after ACS, despite successful PCI revascularization (7–9).

An individualized approach, relying on current clinical guidelines, and applying the appropriate risk scores is needed when deciding on the need for prolonged DAPT. Clinicians are best placed in identifying patients at higher risk of ischaemic events and a low bleeding risk (10, 11).

This study aimed to evaluate the long-term outcomes of extended DAPT therapy following PCI in the context of ACS in real-world practice.

## Methods

### Study design

The study is a retrospective, observational cohort conducted in four hospitals in Madrid, Spain, evaluating patients with ACS, including ST-elevation myocardial infarction (STEMI), non-ST elevation myocardial infarction (NSTEMI) and unstable angina (UA), treated with PCI, and discharged home between January 1, 2017 and December 31, 2018. DAPT with aspirin and either ticagrelor, prasugrel, or clopidogrel was assessed.

### Outcomes and definitions

The primary outcome was a combination of Major Adverse Cardiovascular Events (MACE) and major bleeding events. MACE was defined as a composite of all-cause mortality, non-fatal MI, stent thrombosis and ischaemic stroke. Major bleeding was defined according to the Bleeding Academic Research Consortium (BARC) type 2, 3, or 5 bleeding definitions (12). Secondary outcomes were each component of the composite outcome of MACE: readmission due to angina, new revascularization, target vessel revascularization (TVR), readmission for bleeding and major bleeding and cardiovascular mortality.

TVR was defined as repeated revascularization by PCI or surgery of the target vessel. Myocardial Infarction was defined according to the fourth universal definition of myocardial infarction (13). The definition of duration of DAPT was the length of time between the date of the index PCI procedure and DAPT discontinuation. Patients were classified into two groups according to DAPT duration: ≤13 months and >13 months (since not all the patients had a follow-up visit at exactly 12 months after PCI).

### Study population

The study population was comprised of consecutive patients with DAPT beyond one year following an ACS that was revascularized with stent implantation during the PCI. Inclusion criteria were patients who were discharged following an ACS when they presented obstructive coronary artery disease (defined as stenosis 50% of the left main coronary artery and 70% of any other artery, or invasive evidence of ischemia) diagnosed on the coronary angiography at admission, underwent to PCI with stent implantation, treatment at discharge with DAPT, and survival >1 year after the initial event.

We excluded patients with previous indication for oral anticoagulation (OAC), triple therapy on discharge or indication of OAC during the follow-up, patients with in-hospital mortality or within the first year after index PCI, those with other needs to stop DAPT before 1 year (blood transfusion, urgent surgery or major surgery, oncological pathology that predisposes to a high risk of bleeding). Patients with events as new MI, new revascularization, or major bleeding before 1 year from the index ACS were excluded.

### Study variables and follow-up

For each patient the following information was recorded: demographic, clinical examination findings, laboratory, risk factor assessment, characteristics of coronary disease, treatment at discharge and any history of bleeding. With regards to coronary artery disease, disease of the left main coronary artery (LMCA), proximal left anterior descending artery (LAD), or multivessel disease (MVD) (i.e., at least two coronary territories affected) was documented. The time of DAPT discontinuation was registered during the maximum follow-up period. The documentation of events during follow-up was obtained through an electronic medical record. The ischaemic and bleeding risk profile was assessed during the admission using the following scores: PRECISE-DAPT score (14), DAPT score (15), GRACE score (16) and CRUSADE score (17).

### Data sources

All the variables used in this analysis were obtained from a hospital electronic information system from the Autonomous Community of Madrid containing medical, administrative and laboratory data stored for anonymous data management under the applicable state legislation.

### Statistical analysis

Qualitative variables are presented with their frequency distribution. Quantitative variables are summarized with their mean and standard deviation (SD). Quantitative variables that show a skewed distribution are summarized with the median and the interquartile range (IQR). The *χ*^2^ test or Fisher's exact test was obtained for the comparison of qualitative variables, if necessary. Mean comparisons between the group ≤13 months and >13 months were analysed using Student's *t*-test if the variables follow a normal distribution or using the non-parametric Mann–Whitney *U* test for asymmetric variables. Survival curves were calculated using Kaplan–Meier estimates and compared with the log-rank test. To obtain the crude effect and to adjust for the significant variables of the group of ≤13 months and >13 months on the different definitions of event, Cox regression models were acquired. Rate ratios (Hazard Ratio) are presented as a measure of effect with their corresponding 95% confidence intervals (CI). For all tests, a significance value of 5% was demonstrated. Data processing and analysis were performed using the statistical software IBM SPSS Statistics v.26. The graphics were made using the R Studio v.3.6.0 program.

## Results

**Figure 1:**
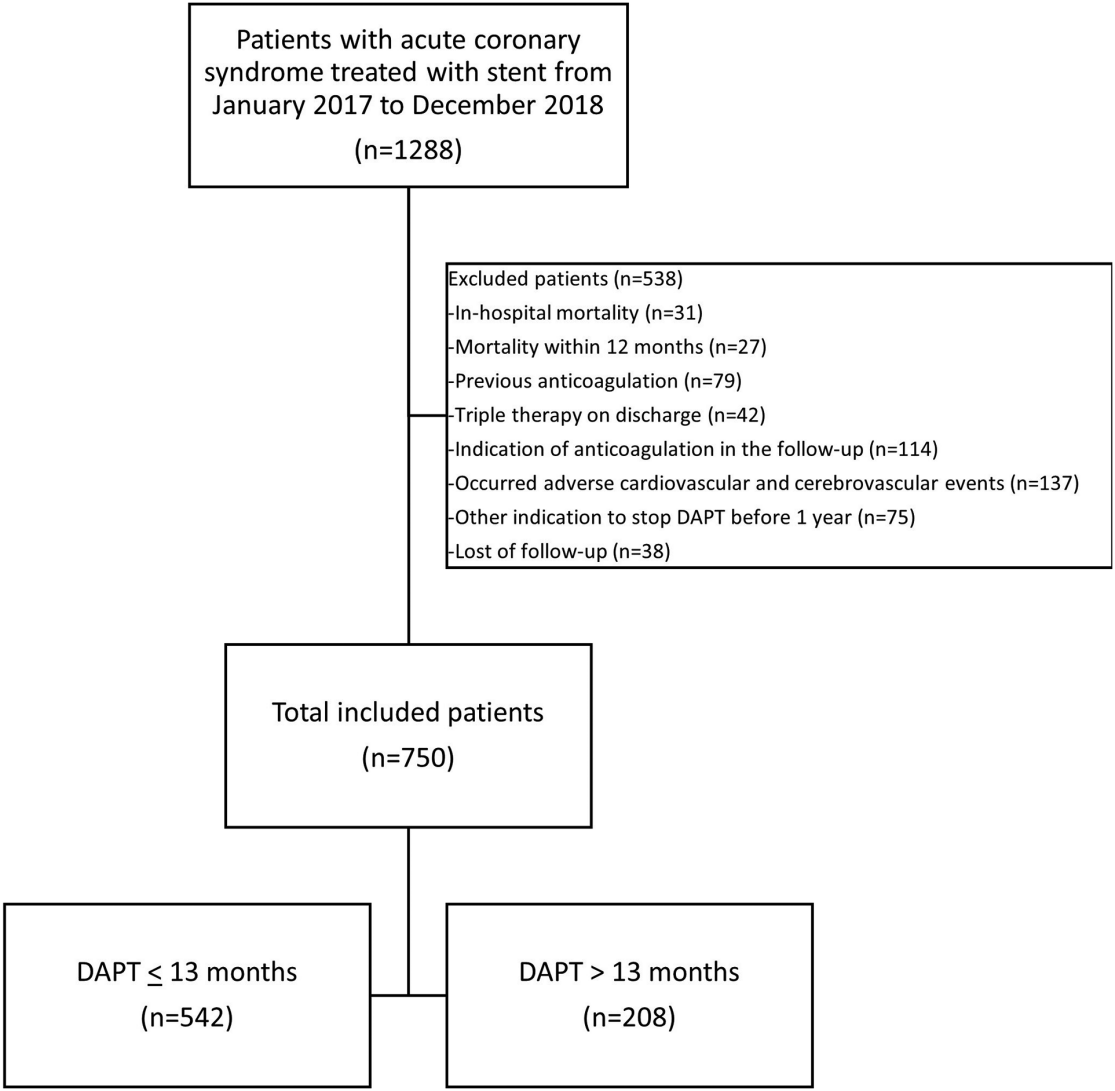
Flowchart of the study.

From a total of 1,288 consecutive ACS patients treated with stent between January 2017 and December 2018, were included 750 patients with complete follow-up (mean follow-up 48 months), 542 patients received DAPT up to 13 months (72.3%). Mean DAPT duration was 370,6 days for ≤13 months group and 765,7 days for ≥13 months group. Figure 1 summarizes the study flow. Excluded patients comprised 58 with in-hospital mortality or within the first year, 235 with indication of OAC, 137 with cardiovascular or cerebrovascular events the first year, and 113 with other indication to stop DAPT or loss of follow-up. Demographic and clinical characteristics are shown in Table 1. Baseline characteristics were similar between the two groups. However, the prevalence of previous coronary artery disease (CAD) was higher in patients with >13 months of DAPT, having a higher percentage of prior MI and prior PCI or coronary artery bypass graft (CABG). Male sex was higher in the group with ≤13 months of DAPT. LMCA stenosis at presentation and multivessel disease were higher in patients who received extended DAPT > 13 months.

**Table 1:** Demographic and clinical characteristics.

	≤13 months (*n* = 542)	>13 months (*n* = 208)	*p*-value
Age (years)	63,5 ± 12,7	65,3 ± 13,2	0.088
Male (%)	444 (81.9%)	155 (74.5%)	**0** **.** **024**
BMI (kg/m^2^)	27.53 ± 4.26	27.46 ± 4.24	0.829
Hypertension	296 (54.6%)	123 (59.1%)	0.264
Diabetes	132 (24.4%)	57 (27.4%)	0.389
Dyslipidaemia	264 (48.7%)	113 (54.3%)	0.168
Smoker	342 (63.1%)	119 (57.2%)	0.138
PAD	34 (6.3%)	9 (4.3%)	0.305
Prior Stroke	25 (4.6%)	16 (7.7%)	0.097
Prior CAD	75 (13.8%)	42 (20.2%)	**0** **.** **032**
Prior MI	48 (8.9%)	31 (14.9%)	**0** **.** **016**
Prior PCI	64 (11.8%)	38 (18.3%)	**0** **.** **021**
Prior CABG	5 (0.9%)	6 (2.9%)	**0** **.** **045**
Congestive Heart Failure	8 (1.5%)	3 (1.4%)	0.973
Prior Bleeding	8 (1.5%)	2 (1.0%)	0.582
Dialysis	2 (0.4%)	4 (1.9%)	0.053
COPD	28 (5.2%)	10 (4.8%)	0.841
Prior major surgery	186 (34.3%)	83 (39.9%)	0.153
Prior statins	179 (33.0%)	94 (45.2%)	**0** **.** **002**
**Presentation:**			
Type of ACS:			0.074
Unstable angina	26 (4.8%)	18 (8.7%)	
STEMI	252 (46.5%)	84 (40.4%)	
NSTEMI	264 (48.7%)	106 (51.0%)	
Killip Class			NS
I	489 (90.2%)	181 (87.0%)	
II	31 (5.7%)	11 (5.3%)	
III	11 (2.0%)	3 (1.4%)	
IV	11 (2.0%)	13 (6.3%)	
HF at presentation	25 (4.6%)	10 (4.8%)	0.910
Cardiac arrest	16 (3.0%)	10 (4.8%)	0.214
Haemoglobin on admission	14.55 ± 1.68	14.16 ± 1.93	**0** **.** **011**
Creatinine on admission	0.98 ± 0.45	1.06 ± 0.68	0.426
GFR (ml/min/1.73m^2^)	91.03 ± 36.61	85.50 ± 36.13	0.062
**Procedural:**			
LMCA stenosis	37 (6.8%)	25 (12.0%)	**0** **.** **021**
Proximal LAD stenosis	146 (26.9%)	69 (33.2%)	0.091
Multivessel disease	322 (59.4%)	141 (67.8%)	**0** **.** **035**
Number of stents	1.46 ± 0.85	1.49 ± 0.81	0.427
Mean length stents (mm)	30.40 ± 20.01	30.17 ± 19.37	0.757
Mean DAPT duration (days)	370.6 ± 28.2	765.7 ± 447.6	–
Mean Follow up (days)	1,456 ± 252	1,453 ± 270	0.893
P2Y12i			0.136
Clopidogrel	104 (19.2%)	48 (23.1%)	
Prasugrel	60 (11.1%)	14 (6.7%)	
Ticagrelor	378 (69.7%)	146 (70.2%)	
LVEF (%)	54.34 ± 7.82	54.20 ± 8.34	0.773
**Scores:**			
DAPT-Score	1.48 ± 1.28	1.41 ± 1.35	0.775
PRECISE-DAPT	17.96 ± 11.74	20.23 ± 12.90	**0** **.** **022**
GRACE	138.6 ± 35.8	143.7 ± 40.7	0.096
CRUSADE	22.2 ± 13.3	24.6 ± 13.9	**0** **.** **028**

BMI, body mass index; PAD, peripheral artery disease; CAD, coronary artery disease; MI, myocardial infarction; PCI, percutaneous coronary intervention; CABG, coronary artery bypass graft; CHF, congestive heart failure; COPD, chronic obstructive pulmonary disease; STEMI, ST-elevation myocardial infarction; NSTEMI, non-ST elevation myocardial infarction; HF, heart failure; GFR, glomerular filtration rate; LMCA, left main coronary artery; LAD, left anterior descending artery; DAPT, dual antiplatelet therapy; LVEF, left ventricular ejection fraction.

Bold values are statistically significant values.

**Figure 2:**
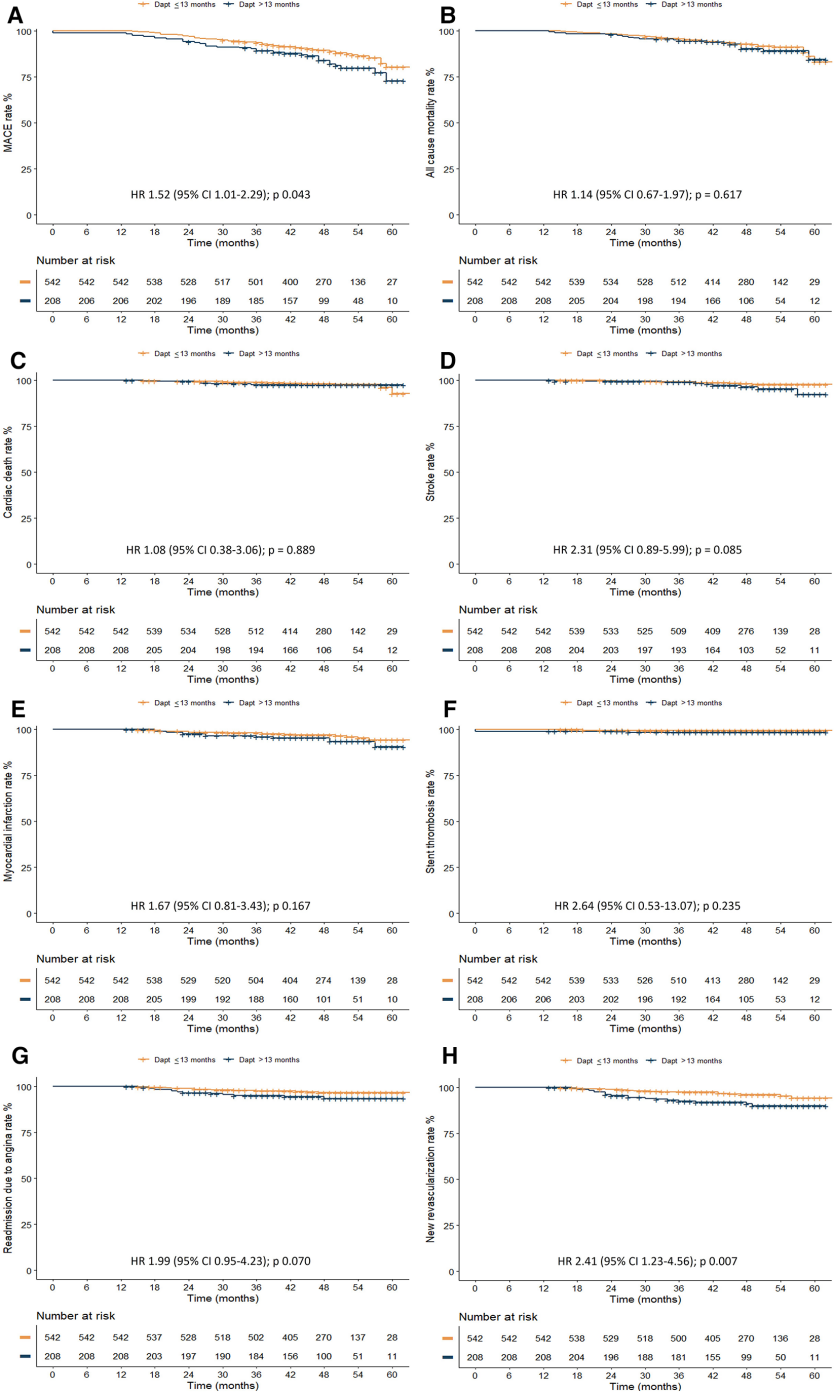
Kaplan–Meier curves for clinical outcomes according to DAPT duration. (**A**) MACE, (**B**) All cause mortality, (**C**) Cardiac death, (**D**) Stroke, (**E**) Myocardial infarction, (**F**) Stent thrombosis, (**G**) Readmission due to angina, (**H**) New revascularization.

During follow-up, patients in the group of DAPT > 13 months had a higher non-adjusted incidence of MACE (11.6% vs. 17.3%, *p* = 0.040) and new revascularization (3.7% vs. 8.7%, *p* = 0.006). Differences in all-cause death (*p* = 0.593), cardiac death (*p* = 1.000), and ischaemic events such as myocardial infarction (*p* = 0.163), stent thrombosis (*p* = 0.356) and stroke (*p* = 0.097) were non-significant (Table 2). There was no difference between groups in terms of incidence of bleeding events, as shown in Table 2. Kaplan–Meier estimates showed the same trend (Figure 2).

**Table 2:** Clinical outcomes in patients in the DAPT ≤ 13 months and >13 months groups during follow-up from the landmark of 12 months of follow-up.

	≤13 months (*n* = 542)	>13 months (*n* = 208)	*p*-value
MACE^a^	63 (11.6%)	36 (17.3%)	**0** **.** **040**
All cause death	43 (7.9%)	19 (9.1%)	0.593
Cardiac death	12 (2.2%)	5 (2.4%)	1.000
Myocardial infarction	19 (3.5%)	12 (5.8%)	0.163
Stent thrombosis	3 (0.6%)	3 (1.4%)	0.356
Readmission due to angina	16 (3.0%)	12 (5.8%)	0.068
New revascularization	20 (3.7%)	18 (8.7%)	**0**.**006**
TVR	11 (2.0%)	9 (4.3%)	0.080
Stroke	9 (1.7%)	8 (3.8%)	0.097
Readmission due to bleeding	7 (1.3%)	2 (1.0%)	1.000
BARC ≥ 2	40 (7.4%)	13 (6.3%)	0.589

Bold values are statistically significant values.

^a^ MACE (Composite of cardiovascular or all-cause death, nonfatal myocardial infarction, stent thrombosis, and stroke). TVR, target vessel revascularization; BARC, bleeding academic research consortium.

Cox proportional hazard model for clinical events showed that patients with DAPT longer than 13 months displayed a higher risk of new revascularization over the observed time (Table 3).

**Table 3:** Cox proportional hazard model for clinical events between groups.

	HR	95% CI	*p*-value
MACE	1.61	(0.99; 2.63)	0.057
All cause death	1.15	(0.67; 1.97)	0.617
Cardiac death	1.08	(0.38; 3.06)	0.889
Stroke	2.31	(0.89; 5.99)	0.085
New Myocardial Infarction	1.67	(0.81; 3.43)	0.167
Stent thrombosis	2.64	(0.53; 13.07)	0.235
Readmission due to angina	1.99	(0.95; 4.23)	0.070
Revascularization	2.41	(1.28; 4.56)	**0** **.** **007**
TVR	2.16	(0.89; 5.21)	0.087
Readmission due to bleeding	0.74	(0.15; 3.58)	0.711
BARC ≥ 2	0.84	(0.45; 1.58)	0.593

Bold values are statistically significant values.

MACE (Composite of cardiovascular or all-cause death, nonfatal myocardial infarction, stent thrombosis, and stroke). TVR, target vessel revascularization; BARC, bleeding academic research consortium.

Univariate Cox regression analysis for MACE is shown in Table 4, showing that the risk factors for MACE were DAPT > 13 months, age, hypertension, diabetes, prior PCI, LM stenosis, multivessel disease, PRECISE-DAPT score, CRUSADE score and GRACE score. The protective factor for MACE was normal haemoglobin (HR: 0.77, 95% CI: 0.70–0.85, *p* < 0.001).

**Table 4:** Cox regression analysis for MACE.

	Univariate		Multivariate^a^	
	HR (95% CI)	*p*-value	HR (95% CI)	*p*-value
DAPT >13 months	1.53 (1.01–2.30)	**0** **.** **043**	1.36 (0.89–2.08)	0.151
Sex (female)	1.08 (0.67–1.73)	0.764	0.68 (0.40–1.14)	0.140
Age	1.05 (1.03–1.07)	**<0** **.** **001**	1.04 (1.02–1.06)	**<0** **.** **001**
BMI	0.97 (0.93–1.02)	0.290		
Hypertension	2.45 (1.56–3.86)	**<0** **.** **001**	1.42 (0.87–2.33)	0.160
DM	2.24 (1.50–3.35)	**<0** **.** **001**	1.46 (0.96–2.23)	0.078
Dyslipidaemia	1.12 (0.75–1.66)	0.584		
Prior MI	1.49 (0.85–2.62)	0.168		
Prior PCI	1.91 (1.18–3.10)	**0** **.** **009**	1.21 (0.73–2.03)	0.459
Prior CABG	0.75 (0.10–5.36)	0.772		
STEMI vs. NSTEMI	1.41 (0.93–2.12)	0.104	0.99 (0.65–1.52)	0.969
LMCA stenosis	1.89 (1.07–3.32)	**0** **.** **028**	0.84 (0.46–1.54)	0.565
Proximal LAD stenosis	0.89 (0.63–1.50)	0.886		
Multivessel disease	3.14 (1.86–5.30)	**<0** **.** **001**	2.26 (1.31–3.91)	**0** **.** **003**
Number of stents	1.09 (0.88–1.35)	0.419		
Length of stents	1.01 (0.99–1.01)	0.263		
Haemoglobin	0.77 (0.70–0.85)	**<0** **.** **001**	0.88 (0.79–0.99)	**0** **.** **034**
DAPT-score	0.94 (0.81–1.09)	0.418		
PRECISE-DAPT	1.04 (1.03–1.05)	**<0** **.** **001**		
CRUSADE	1.05 (1.03–1.06)	**<0** **.** **001**		
GRACE	1.01 (1.01–1.02)	**<0** **.** **001**		

DAPT, dual antiplatelet therapy; BMI, body mass index; DM, diabetes mellitus; MI, myocardial infarction; PCI, percutaneous coronary intervention; CABG, coronary artery bypass graft; STEMI, ST-elevation myocardial infarction; NSTEMI, non-ST elevation myocardial infarction; LMCA, left main coronary artery; LAD, left anterior descending artery.

Bold values are statistically significant values.

^a^ Multivariate analysis adjusted to clinically relevant variables.

Multivariable Cox regression analysis showed that the independent risk predictors of MACE were age (HR: 1.04, 95% CI: 1.02–1.06, *p* < 0.001) and multivessel disease (HR: 2.29, 95% CI: 1.32–3.95, *p* = 0.003), whereas the independent protective predictor was normal haemoglobin (HR: 0.88, 95% CI: 0.78–0.98, *p* = 0.022) (Table 4).

The ischaemic and bleeding risk profiles of our patients, assessed during the admission with the PRECISE-DAPT score (14), DAPT score (15), GRACE score (16) and CRUSADE score (17), were similar in both groups, with the exception of CRUSADE score and PRECISE-DAPT score (Table 1), with higher scores with prolonged therapy.

## Discussion

In this “real-world practice” registry of patients treated with PCI in the context of an ACS, who survived the first year uneventfully following the initial presentation, we analysed the DAPT duration pattern beyond one year with a mean follow-up time of 48 months. Our main findings were an increase in long-term MACE and new revascularization, without clear differences in other ischaemic events, mortality events, or bleeding events in the >13 months DAPT cohort.

These higher event rates may be explained by the fact that in the group that extended the DAPT > 13 months, there was a more complex CAD, with higher rates of previous PCI or CABG. Additionally, at index presentation, this group had a higher percentage of LMCA stenosis or MVD, which means higher residual risk for ischaemic events.

These results are in contrast to other reports on short vs. extended DAPT, which report a decrease in ischaemic events with higher bleeding rates (18), no differences in ischaemic events with an increased risk of bleeding (19–21) or no significant differences between ischaemic and bleeding events (22–24). Nevertheless, these trials included patients with acute or chronic coronary syndromes, and elective PCI.

Our registry is an observational-retrospective design in ACS patients, nevertheless, large randomized trials in the specific setting of ACS, as the DAPT trial reported reduced ischaemic events but increased bleeding compared with treatment with aspirin alone (25). PEGASUS-TIMI 54 trial showed a reduced risk of cardiovascular death, myocardial infarction, or stroke and an increased risk of TIMI major bleeding using ticagrelor (26). Similar results were reported with the use of clopidogrel for more than 12 months in patients receiving DES (27). In addition, another Chinese study reported lower mortality rates and MACE, less stent thrombosis, and no differences in bleeding events (9). A meta-analysis reported a short duration of DAPT may be safe, with similar rates of thrombotic events and mortality, but exhibited higher rates of haemorrhagic complications in those with extended DAPT duration (28). A recent publication with a similar population to ours, showed no differences in MACE, all-cause mortality or stroke, and increased rates of major bleeding in patients who extended DAPT, with a risk difference of 4.1% between groups (5).

A Chinese trial showed higher rates of MACE in the >1-year DAPT group, similar to our findings. However, unlike our report, their incidence of all-cause death, cardiac death and stent thrombosis in the 1-year DAPT group was higher, with higher rates of TVR in the prolonged DAPT group, with no differences in bleeding events (1). Finally, the patient profile likely augments the response to the treatment and differences in DAPT outcomes are, at least somewhat explained by different comorbidities, therefore we could only partially adjust for this in our study given its observational design. This reenforces the importance of carefully individualizing the duration of DAPT in the patients we treat daily.

In our population, we displayed patients with a prior history of CAD, MI, previous PCI or CABG, LMCA stenosis, and MVD tended to receive DAPT during >13 months; this is consistent with previous reports on the determinants of extended DAPT (8, 29). This is a relevant feature to consider.

Prediction scores and practice guidelines have been published to help inform this clinical decision-making process for DAPT use ≥1 year post-MI (29). However, their accuracy and discriminative power for identifying patients who would benefit from DAPT for >12 months have not been consistently demonstrated (30). In our population, despite the significant differences between groups in the PRECISE DAPT and CRUSADE scores, there was no increase in bleeding events.

Our report is one of the first real-world, long-term outcomes trials reported in this clinical setting in Spain, as well as the first reporting a higher rate of new revascularization not associated with TVR in ACS patients, different to previous reports. Our findings may suggest that in high-risk patients, prolonged DAPT may not be sufficient to completely reduce their long-term risk; ACS patient profile and its high atherosclerosis burden must be taken into account for improved optimization of the other pharmacological therapies. Contrastingly, no clear higher risk of clinically relevant bleeding was found despite theoretical higher bleeding risk based on the patients CRUSADE score.

## Limitations

Our study has several limitations. This is an observational study with intrinsic limitations related to its retrospective design. Therefore, the results should be interpreted cautiously and only considered hypothesis-generating. However, although the duration of DAPT was not randomized, it was individualized at the treating physician discretion; therefore, our findings reflect real-world practice. The evaluation of drug therapy efficacy is limited. We included ACS patients who remained event-free during the first 12 months following PCI. This study may not apply to patients who undergo bypass surgery or receive medical treatment. Our data were obtained from four centres, however, did not assess all patients in Spain. Larger studies are required to demonstrate these findings with adequate power.

## Conclusions

In this real-world registry cohort of ACS patients treated with PCI and 1 year of DAPT, patients undergoing prolonged DAPT compared to those only treated with aspirin monotherapy had a trend of increased risk of MACE and further revascularization, without differences in other ischaemic or major bleeding events. Despite the current evidence, our findings support the need for future randomized controlled trials to confirm or refute these results in the Spanish population.

## Data Availability

The raw data supporting the conclusions of this article will be made available by the authors, without undue reservation.
